# The Proteome of the Red Blood Cell: An Auspicious Source of New Insights into Membrane-Centered Regulation of Homeostasis

**DOI:** 10.3390/proteomes4040035

**Published:** 2016-11-25

**Authors:** Giel J. C. G. M. Bosman

**Affiliations:** Department of Biochemistry (286), Radboud University Medical Center and Radboud Institute for Molecular Sciences, P.O. Box 9101, NL-6500 HB Nijmegen, The Netherlands; Giel.Bosman@radboudumc.nl

**Keywords:** aging, biomarkers, metabolomics, membrane, proteomics, red blood cells

## Abstract

During the past decade, the hand-in-hand development of biotechnology and bioinformatics has enabled a view of the function of the red blood cell that surpasses the supply of oxygen and removal of carbon dioxide. Comparative proteomic inventories have yielded new clues to the processes that regulate membrane–cytoskeleton interactions in health and disease, and to the ways by which red blood cells communicate with their environment. In addition, proteomic data have revealed the possibility that many, hitherto unsuspected, metabolic processes are active in the red blood cell cytoplasm. Recent metabolomic studies have confirmed and expanded this notion. Taken together, the presently available data point towards the red blood cell membrane as the hub at which all regulatory processes come together. Thus, alterations in the association of regulatory proteins with the cell membrane may be a sine qua non for the functional relevance of any postulated molecular mechanism. From this perspective, comparative proteomics centered on the red blood cell membrane constitute a powerful tool for the identification and elucidation of the physiologically and pathologically relevant pathways that regulate red blood cell homeostasis. Additionally, this perspective provides a focus for the interpretation of metabolomic studies, especially in the development of biomarkers in the blood.

## 1. Introduction

During the past decade, the hand-in-hand development of biotechnology and bioinformatics has enabled a view of the function of the red blood cell that surpasses the supply of oxygen and removal of carbon dioxide, as well as the known build up of its membrane that underlie its unique deformability. Both are textbook examples of molecular structure–function relationships, and of the mechanisms of red blood cell-centered pathologies such as sickle cell disease and spherocytosis. Proteomic inventories of the red blood cell membrane have generated detailed qualitative and semi-quantitative comparisons of pathological with physiological red blood cells, without the restrictions or bias imposed by the detection possibilities imposed by immunoblotting or flow cytometry. Such comparative proteomic inventories have yielded new clues to the processes that regulate membrane–cytoskeleton protein interactions, and to the ways by which red blood cells communicate with their environment, such as with the immune system during cellular aging in vivo. In addition, proteomic data have revealed the possibility that many, hitherto unsuspected, metabolic processes are active in the hemoglobin-dominated cytoplasm. More recently, metabolomic data have confirmed and expanded this notion. In this review, I have selected some data to illustrate these statements, with the goal to sketch their implications for the view of the red blood cell as an important and hitherto sometimes underestimated factor in organismal metabolism.

## 2. The Red Blood Cell Membrane

All published red blood cell membrane proteomes contain, in addition to the well-known, widespread structural constituents of the membrane–cytoskeleton complex, many proteins that are associated with alterations in protein conformation, post-translational modifications, or both. Some of these proteins are likely to be recruited from the cytosol to the membrane in response to the isolation of red blood cells, the removal of hemoglobin, the purification of the membrane fraction, or any combination of these [1]. However, the relatively high numbers of some of them in various proteomes obtained by different isolation methods suggest, in general, physiological roles ([1], and references therein). The exact nature of these roles awaits identification of their binding partners and of the triggers for their binding, as already suggested in one of the first comprehensive inventories of the red blood cell proteome [2]. It also remains to be established if this recruitment is permanent, and if so, whether it happened during erythropoiesis or during the cell’s sojourn in the circulation. These are not trivial questions, as the answers are likely to reveal pathways in the regulation of function, aging and survival, in response to the molecules and cells that red blood cells encounter when traveling through the body.

Similarly, comparative proteomics is rapidly expanding our knowledge on the mechanisms underlying pathological red blood cell shape [3,4]. For example, the presence of the active form of the protein kinase Lyn in the membrane fractions of acanthocytes confirms and expands the role of reversible phosphorylation in the interaction between integral membrane and cytoskeleton proteins, as had been indicated by phosphoproteomic data [5,6]. Classical analysis using membrane protein staining and immunochemical methods could reveal only subtle changes in band 3 conformation in these cells [7]. The use of semi-quantitative, comparative proteomics, however, revealed a cell shape-associated increase in the association of stomatin, proteins of the small G protein family, and the ankyrin complex with the lipid bilayer [4]. These data illustrate the powerful combination of proteomics analysis with more specific mass spectrometric analysis of posttranslational modifications in the elucidation of pathology-associated changes in the structure of the red blood cell membrane. Within this membrane, a dynamic interaction between lipid bilayer and cytoskeleton enables appropriate, and sometimes extreme, deformation of the red blood cell in the circulation. Thus, detailed information, in combination with the description of an increasingly refined interactome, will contribute to a better understanding of this essential function [8,9,10]. Developments such as these have, in a relatively short time, generated a wealth of information on the changes in the red blood cell proteome during red blood bank storage. Amongst others, proteomic data have confirmed and extended the central role of band 3, together with the activation of protein-protecting and protein-removing mechanisms, in the storage-accompanying changes in metabolism, morphology, and function [11,12,13].

Phosphorylation-dependent association of key glycolytic enzymes with band 3 is part of the oxygen concentration-triggered regulation of ATP production and redox status, that may also regulate cell shape and deformability [14]. Data from labeling studies and pharmacological interventions in vitro suggest that a relationship between membrane organization and cytoplasmic protein association may not be restricted to band 3 and key glycolytic enzymes [15,16]. The signals that affect this association, their receptors, and their transduction pathways are largely unknown. Additionally, such signals may regulate the activity of the many membrane transporters that have been identified in the red blood cell proteome. In patients with hemoglobinopathies or membranopathies, altered transport of selected metabolites may be part of the pathophysiology [17,18]. The elucidation of these signaling networks constitutes a major, exciting challenge for the next decade. The relevance of this endeavor is rapidly expanding with the large-scale search for biomarkers, and the concomitant increase in the awareness of the quantitative as well as qualitative importance of the red blood cell compartment of the blood.

## 3. The Red Blood Cell Cytoplasm

Proteome analysis of the red blood cell soluble fraction has not only shown the presence not only of the enzymes of the metabolic pathways known to be active in the red blood cell, such as the glycolysis and the pentose phosphate pathways, but also of enzymes involved in the protection against oxidative damage [1,2,19,20,21,22]. Additionally, red blood cells contain an abundance of proteins involved in the “repair or destroy” of damaged proteins [8,22]. Proteins of this category are heat shock proteins and chaperones, and proteases and proteasome components. It is striking that these enzymes seem to be organized in interacting multiprotein complexes, suggesting an intensive cross-talk between oxygen transport, metabolism, anti-oxidant activity, and protein breakdown [23,24]. Recent data have not only expanded the original models on the oxygen-dependent modulation of the red blood cell metabolome [25,26], but may also link the concentration of carbon dioxide, independent of its effect on intracellular pH, to the activity of these complexes [27]. The unexpected finding of a reduction in transketolase, an enzyme of the pentose phosphate pathway, in these same red blood cells [28] represents an illustration of the power of proteomic analysis in the unearthing of the cellular “interactome” [8,10,23].

Many cytoplasmic proteins are reduced in number by enucleation and vesiculation during maturation of reticulocytes [29,30]. In addition, the process of aging-associated vesiculation results in the specific removal of both cytosplasmic proteins such as modified hemoglobins and damaged membrane proteins such as band 3 [31]. During the vesiculation that is part of the reticulocyte maturation process, nuclear and ribosomal proteins end up in the pyrenocyte, whereas proteins with “repair or destroy” functions are concentrated in the erythrocyte [29,30]. These studies also show that, although many cytosolic proteins are reduced in number during maturation, many proteins in the cytoplasm of the mature red blood cell may very well be the remnants of metabolic pathways that are no longer functional. Notable exceptions are likely to be the many proteins involved in lipid binding or metabolism, which constitute the largest proportion of proteins that are higher in adult red blood cells than in reticulocytes, even larger than the oxygen-binding proteins [29,32].

## 4. The Red Blood Cell Metabolism

With each new proteomic inventory of the membrane protein composition of the red blood cell, there is an exponential increase in the need for translation of this information into implications for function and homeostasis. From the beginning, this has inspired research on a phosphorylation-centered regulation of protein–protein interactions, especially in misshapen and stored red blood cells [5,6,12]. Comparative inventories of the red blood cell metabolome constitute the next phase in the translation of the proteomics data into functional information on the red blood cell metabolism. The close relationship between oxygen binding, ATP production, regulation of pH and redox status, and deformability [14] by itself warrants any attempt to a better understanding of its molecular foundation. Metabolomic data point towards genetically determined heterogeneity in the proteome, as apparent from the deduced activity of key enzymes of glycolysis and redox homeostasis [33]. Proteomic as well as metabolomic data suggest that other metabolic pathways have remained underexposed or neglected, such as the metabolism of amino acids, nucleotides, cofactors, and bioactive as well as structural lipids [29,32,33]. These pathways are likely to receive more attention in the near future. One reason is the rapid development of the application of metabolomics in the development of biomarkers and pharmacometabolomic signatures [34]. Another reason is the growing awareness of the effect of systemic disease states on red blood cell survival, as exemplified in patients with anemia of chronic disease and anemia of inflammation [35,36].

## 5. Proteomics and Red Blood Cell Homeostasis: Signaling and Regulation Centered at the Cell Membrane

The data presented above sketch a picture from which molecular interactions at the red blood cell membrane emerge as the main organizing forces, which regulate not only cell shape and deformability, but also metabolism, and thereby oxygen and carbon dioxide transport as well as cell survival. This may be obvious for proteins with an origin in the plasma; the presence of immunoglobulins and complement factors in the membrane proteome is likely to be the result of their binding to damaged and/or senescent red blood cells in the population [22,23]. Additionally, changes in the interaction between integral membrane proteins and cytoskeletal components may reveal the mechanisms underlying physiological or pathological changes in red blood cell volume and morphology. For example, the aging-related decrease in the spectrin and ankyrin content, together with the apparent enrichment of actin in the membranes of red blood cells aged in vivo, support a band 3-centered aging mechanism [37]. Such proteomic data give directions for future research on the underlying molecular changes that lead to the identity of the physiological removal signals, and to the mechanisms of vesiculation in healthy subjects and in patients with red blood cell-affecting diseases [13,22]. Subsequent identification of the aging-associated modifications is likely to enable the long-due step from association to causation. 

Additionally, proteomic data have indicated that chaperones and heat shock stress proteins may become enriched in the red blood cell membrane. Such recruitment is likely to be the consequence of the, probably oxidation-induced, unfolding and exposure of hydrophobic stretches of membrane proteins [22,23]. The presence of regulatory networks, involving receptor-mediated signaling, constitutes a more complex conceptual challenge. Various receptors have been identified in the membrane fraction, as well as activated forms of kinases and secondary messengers [38,39]. Already, a combination of refined cell age separation techniques with the measurement of phosphatidylserine exposure has indicated the involvement of hitherto unsuspected signaling pathways in the recognition and removal of damaged red blood cells [40]. Observations such as these confirm and expand the hypotheses inspired from the proteomic inventories of red blood cells aged in vivo or in vitro [21,22,41]. It has been shown that oxygen-regulated association of key enzymes with the membrane regulates the activity of glycolysis and the pentose phosphate pathway, and that oxygen-mediated association of ankyrin with band 3 affects the binding between the cytoskeleton and the lipid bilayer [14,15,42]. Similarly, the association of proteins such as G protein subunits and activated forms of phosphorylating enzymes with membrane components, possibly linked to calcium-related signaling [43], are likely to constitute essential roles in the signaling networks regulating red blood cell homeostasis [5,22]. In recent studies on the association of metabolic changes with red blood cell-centered diseases, some changes in the metabolome were clearly associated with the pathophysiology, as indicated by the increased concentrations of malate and various amino acids in HbS cells [18]. On the other hand, GSH and GSSG concentrations and acetyl-carnitine were found to be associated with the red blood cell aging process [17,18]. In both cases, alterations in membrane protein composition and organization are likely to be the primary cause of at least some of these changes [16,44]. 

## 6. Conclusions

I argue here that, in the elucidation of the pathways involved in red blood cell-centered homeostasis, alterations in the association of regulatory proteins with the cell membrane constitute a sine qua non for the functional relevance of any postulated molecular mechanism. From this perspective, comparative proteomics provide a powerful tool for the identification and elucidation of the pathways that regulate red blood cell homeostasis in health and disease. This perspective may be valuable for the interpretation of blood-centered metabolomic studies, especially for the development of sensitive and specific biomarkers in the blood.
